# Multi-omics analysis unveils inflammatory and metabolic alterations between the synovium and cartilage in patients with knee osteoarthritis

**DOI:** 10.3389/fmolb.2026.1855139

**Published:** 2026-09-22

**Authors:** Wenlong Xu, Shao Cheng, Zhen Wang, Shang Ma, Xiaokun Dong, Jinxin Ma, Bowen Zheng, Bingqi Wei, Shangzeng Wang

**Affiliations:** 1 College of Orthopedics and Traumatology, Henan University of Chinese Medicine, Zhengzhou, China; 2 Department of Joint Diseases, Henan Provincial Hospital of Traditional Chinese Medicine (Second Affiliated Hospital of Henan University of Chinese Medicine), Zhengzhou, China

**Keywords:** knee osteoarthritis, metabolic exhaustion, multi-omics, synovial inflammation, TIAM1-Rac1 signaling

## Abstract

**Background:**

KOA (knee osteoarthritis) is a degenerative disease affecting the entire joint, involving synovial inflammation and cartilage degeneration, but their molecular interplay remains unclear. This study integrated proteomics and metabolomics to investigate cross-tissue alterations in KOA.

**Methods:**

Paired synovial and cartilage tissues from 12 KOA patients and 12 controls were analyzed using DIA proteomics and untargeted metabolomics. The major molecular abnormalities in OA synovium and cartilage were evaluated through differential expression analysis, GO and KEGG functional enrichment analyses, protein–protein interaction network analysis, protein–metabolite integrated analysis, and cross-tissue integrative analysis. TIAM1, Rac1, and MRPS7 were validated by Western blot.

**Results:**

Proteomic analysis showed that proteins related to inflammation and cytoskeletal remodeling were significantly upregulated in OA synovial tissue, including increased expression of TIAM1. In cartilage tissue, proteins related to mitochondrial translation and energy metabolism were significantly downregulated, including decreased expression of MRPS7. Metabolomic analysis indicated enhanced arachidonic acid metabolism in synovial tissue, with significantly increased levels of inflammation-related metabolites such as PGE2 and 12-HETE. In cartilage tissue, metabolites related to the tricarboxylic acid cycle and pyrimidine metabolism were decreased, suggesting impaired energy supply and nucleotide metabolism. Protein–metabolite integrated analysis further showed that the differential molecules in OA synovium were mainly involved in inflammatory signaling, nucleotide metabolism, carbohydrate metabolism, and lipid mediator output, whereas those in OA cartilage were mainly associated with mitochondrial function, basal metabolism, cellular stress, and matrix homeostasis-related processes. Integrated analysis suggested that the synovial TIAM1/Rac1-related pathway may be involved in the inflammatory microenvironment and cytoskeletal remodeling, whereas the downregulation of MRPS7 in cartilage indicates suppressed mitochondrial translation. Western blot results further validated the upregulation of TIAM1 and Rac1 in OA synovium and the downregulation of MRPS7 in OA cartilage.

**Conclusion:**

This multi omics study revealed the cross tissue molecular characteristics of KOA, manifested as synovitis metabolic activation and cartilage energy metabolism dysfunction, suggesting that it may be closely related to the regulation of TIAM1-Rac1 signaling axis and mitochondrial damage. These findings provide a new perspective for a deeper understanding of the pathogenesis of KOA and point out potential therapeutic intervention targets.

## Background

KOA (knee osteoarthritis) is among the most prevalent degenerative joint diseases encountered in clinical practice. It is primarily characterized by the degeneration of knee cartilage, an inflammatory response in the synovium, subchondral bone remodeling, and the formation of osteophytes. The incidence of KOA significantly increases with age, making it a leading cause of disability in the elderly population (Glyn-Jones et al., 2015). Epidemiological studies indicate that the global prevalence of KOA affects approximately 10%–15% of middle-aged and elderly individuals, with rates rising annually. This trend has a profound impact on patients’ quality of life and imposes a substantial burden on the healthcare system (Courties et al., 2024). Initially, KOA was perceived as a “mechanical wear” disease, with research predominantly focusing on mechanical factors such as age, trauma, and cartilage degeneration in elucidating its pathogenesis (Gelber, 2024). The prevailing hypothesis regarding the pathogenesis of OA (osteoarthritis) suggests that the disease typically initiates with damage to the joint structure resulting from biomechanical factors. This damage triggers the release of various biological mediators, which subsequently activate multiple inflammatory pathways, ultimately leading to progressive cartilage tissue deterioration (Sanchez-Lopez et al., 2022). Radiological assessments commonly reveal significant features, including narrowed joint space, osteophyte formation, and subchondral osteonecrosis. These manifestations often indicate that KOA has advanced to a later stage, suggesting that the optimal treatment window has been missed (Kellgren and Lawrence, 1957). However, recent research indicates that the pathogenesis of KOA is considerably more intricate than mere mechanical wear and tear; it is a systemic disease that encompasses the synovial membrane, cartilage, subchondral bone, and immune system (Loeser et al., 2016; Liu B. et al., 2024).

Synovitis has been widely recognized as a significant driving force in the pathological progression of KOA (Wang et al., 2022; Neogi et al., 2016). The normal synovial membrane primarily consists of synovial cells and a limited number of immune cells, playing a crucial role in maintaining joint fluid homeostasis. In contrast, KOA is characterized by a chronic inflammatory activation of the synovium, which is evident through synovial hyperplasia, angiogenesis, and infiltration of inflammatory cells. Synovial cells release substantial quantities of pro-inflammatory cytokines, including IL-1β, TNF-α, and IL-6, as well as chemokines, thereby activating downstream signaling pathways such as NF-κB and MAPK (Zhang et al., 2018a; Philpott et al., 2025). This process accelerates chondrocyte apoptosis and matrix degradation. Furthermore, exosomes, metabolites, and oxidative stress factors released by the synovial membrane can traverse the synovial fluid barrier and influence chondrocytes, leading to an imbalance in energy metabolism and mitochondrial dysfunction, which further exacerbates cartilage degeneration. Research indicates that the extent of synovitis is closely associated with the KOA pain score and the severity of the disease. Notably, synovitis can be detected even in the early stages when no significant cartilage damage is evident on imaging (Hill et al., 2007). Consequently, synovitis is considered a critical target for the early diagnosis and intervention of KOA. Numerous studies have demonstrated the presence of various differentially expressed proteins and metabolites in the synovium of KOA, highlighting the significant roles of inflammatory responses, matrix remodeling, and energy metabolism disorders in the disease’s pathogenesis. A range of extracellular matrix-related proteins has been found to be significantly upregulated in the synovial tissue of KOA. Fibronectin (FN1), an essential adhesion glycoprotein in the extracellular matrix, regulates cell migration, proliferation, and signal transduction. Its upregulation is thought to be closely associated with the persistence of synovial fibrosis and inflammation. Additionally, fibronectin fragments, resulting from FN1 proteolysis, serve as critical pathogenic mediators in the development of KOA by preferentially inhibiting proteoglycan biosynthesis in articular chondrocytes, thereby disrupting cartilage homeostasis and promoting tissue degeneration (Dang et al., 2003). TGFBI, a downstream effector of the TGF-β signaling pathway, promotes fibroblast differentiation and plays a role in extracellular matrix (ECM) remodeling. The elevated levels of COL1A1 indicate that synovial tissue is predisposed to fibrosis in a chronic inflammatory environment, aligning with the pathological features of thickening, vascularization, and collagen deposition observed in the synovial tissue of KOA patients (Ge et al., 2025).

During the progression of KOA, chondrocytes undergo various morphological and functional alterations, often characterized by the coexistence of necrosis and increased metabolic activity. In patients with KOA, the structural arrangement of collagen fibers within the cartilage matrix is disordered, accompanied by abnormal deposition of calcium salt crystals (Schelbergen et al., 2016). Research indicates that joint inflammation signals, such as IL-1α, significantly upregulate the expression of the mechanosensitive ion channel Piezo1 in chondrocytes via the p38 MAPK pathway and transcription factors (ATF2/CREBP1, HNF4). Elevated levels of Piezo1 result in increased resting calcium ion concentrations and heightened mechanosensitivity in cells. This condition further disrupts the cytoskeleton, characterized by F-actin sparsity, rendering chondrocytes more susceptible to damage under mechanical stress (Brylka et al., 2024; Lee et al., 2021). In KOA, chondrocyte metabolic dysfunction is characterized by increased apoptosis, diminished matrix synthesis, and heightened degradation. Inflammatory factors and oxidative stress can induce the overexpression of matrix metalloproteinases (MMPs) and members of the ADAMTS family (Tavallaee et al., 2022; Son et al., 2017), thereby accelerating the degradation of the ECM (Naba, 2024). Concurrently, the imbalance in energy metabolism within chondrocytes, along with abnormal glycolysis and lipid metabolism, is thought to be closely associated with chondrocyte aging (Zheng et al., 2021). Recent studies indicate that alterations in chondrocyte metabolism are not only a consequence but also likely precursors of inflammatory responses and disruptions in cellular signaling pathways. In OA, chondrocytes exhibit reprogrammed energy metabolism, characterized by enhanced glycolysis, which results in increased lactic acid production, and compromised mitochondrial oxidative phosphorylation, leading to reduced TCA cycle flux and insufficient ATP production. This metabolic dysfunction contributes to abnormal chondrocyte activity, and short-term inhibition of glycolysis has been shown to diminish the catabolic function of cartilage within an inflammatory context. Thus, targeting energy metabolism presents a promising therapeutic strategy for OA (Zheng et al., 2021; Wu et al., 2023).

The core pathological process of KOA involves a close pathological linkage between the inflammatory response of synovial tissue and the degenerative changes in articular cartilage. These two processes exacerbate one another, creating a vicious cycle that drives disease progression. Pro-inflammatory cytokines and exosomes produced by the synovium can activate the NF-κB and JAK/STAT pathways in chondrocytes, thereby promoting the expression of degrading enzymes. Additionally, matrix degradation fragments released from damaged cartilage, including COL2A1 fragments and hyaluronic acid oligosaccharides, can reactivate synovial macrophages and fibroblasts, leading to the secretion of increased inflammatory mediators (Liu B. et al., 2024; Yang et al., 2024). This feedback loop involving “inflammation - metabolism - matrix” causes KOA to display persistent and progressive characteristics of chronic degeneration. Consequently, investigating the signaling interactions between the synovium and cartilage is essential for elucidating the systemic pathogenesis network of KOA and for establishing a foundation for combined targeted therapies.

KOA, a prototypical generalized degenerative disease, encompasses multiple processes, including synovial inflammatory responses, chondrocyte metabolic disorders, and extracellular matrix remodeling during its onset and progression. Prior research has predominantly concentrated on investigations at the level of individual tissues or specific omics, often focusing solely on the proteomic and metabolomic characteristics of tissues such as the synovium, meniscus, or subchondral bone (Tan et al., 2024; Park et al., 2021). While numerous differential molecules associated with KOA have been identified, there remains a significant gap in the comprehensive analysis of signaling interactions and systemic mechanisms across various tissues. This study aims to address the identified research gap by collecting synovial tissue and articular cartilage samples from both healthy individuals and patients with KOA. It will employ proteomics and metabolomics sequencing to analyze these samples. By comparing the differential expression profiles across the various groups, the study will identify key differential proteins and metabolites. Furthermore, it will utilize Gene Ontology (GO) and Kyoto Encyclopedia of Genes and Genomes (KEGG) pathway enrichment analyses, along with protein-protein interaction (PPI) network analysis, to examine the functional roles of these molecules in biological processes such as inflammatory response, extracellular matrix metabolism, energy metabolism, and oxidative stress. Additionally, this research will compare the molecular characteristics of the synovium and cartilage, investigate potential shared pathways and regulatory nodes involved in the pathogenesis of KOA, and construct an interaction network encompassing “synovium-cartilage-inflammation-metabolism” to elucidate the systemic pathological mechanisms underlying KOA. This study employs a joint approach of proteomics and metabolomics to identify potential early diagnostic biomarkers. Additionally, it offers a novel molecular basis for understanding the pathological progression of KOA and for identifying common therapeutic targets. Consequently, this research establishes a theoretical foundation for precise classification and targeted intervention strategies.

### Ethical approval

The study protocol was reviewed and approved by the Ethics Committee of Henan Provincial Hospital of Traditional Chinese Medicine (The Second Affiliated Hospital of Henan University of Chinese Medicine), Zhengzhou, China. (Approval No. AF/SC-04/05.0; Date: 25 December 2025). A total of 12 patients with KOA were enrolled in the experimental group, and 12 non-arthritic control individuals undergoing surgery for non-degenerative orthopedic conditions were enrolled as the control group. All participants provided written informed consent prior to surgery. Preoperative diagnosis, surgical procedures, arthroscopic sample collection, and final diagnosis were performed by the same medical team. All participants were followed up, and their baseline characteristics are summarized in Table 1. All experimental procedures were conducted in strict accordance with the Declaration of Helsinki and relevant ethical guidelines.

**TABLE 1:** Comparison of clinical characteristics between KOA patients and controls.

Characteristic	Control (n = 12)	OA (n = 12)	P value
Age (years, x ± s)	52.8 ± 11.2	66.3 ± 8.5	0.006
Male, n (%)	5 (41.7%)	4 (33.3%)	0.673
BMI(kg/m^2^, x ± s)	24.1 ± 2.6	27.2 ± 3.3	0.018
Disease duration (years, x ± s)	—	6.8 ± 3.9	—
Kellgren-Lawrence grade (II/III/IV), n	—	3/6/3	—
Pain VAS score (x ± s)	1.3 ± 0.9	6.9 ± 1.6	<0.001
Lysholm knee score (x ± s)	91.8 ± 4.5	51.6 ± 8.9	<0.001

## Methods

### Inclusion criteria

KOA group: Patients who met the clinical and radiographic diagnostic criteria for KOA established by the American College of Rheumatology (ACR); presented with end-stage manifestations according to Kellgren-Lawrence (K-L) grading and MRI findings; exhibited failure of conservative treatment and fulfilled the surgical indications for unicompartmental knee arthroplasty or total knee arthroplasty; had no severe systemic diseases; and had not received any medications affecting metabolic or inflammatory status (e.g., glucocorticoids, immunosuppressants) for at least 3 months prior to enrollment.

Control group: Individuals who underwent surgery for non-degenerative conditions such as extra-articular fractures, lower limb amputation, isolated meniscal injury, or congenital discoid meniscus; exhibited no evidence of inflammatory synovium or synovial fluid upon intraoperative exploration, allowing for the collection of normal synovial and cartilage tissues; had no history of knee pain, mobility limitations, or radiographic signs of degeneration; had available clinical information for comparison with the KOA group; and had no concurrent inflammatory, metabolic, or immune system diseases.

All participants were initially evaluated by orthopedic specialists with 5–20 years of clinical experience. Comprehensive clinical characteristics, including the degree of knee pain, range of motion, joint effusion, and deformity, were systematically recorded through medical history taking, specialized examinations, and physical assessments. Radiographic evaluations were independently performed by two radiologists with intermediate seniority or higher and 5–10 years of diagnostic experience. The degree of cartilage degeneration, synovial hypertrophy, and joint space alterations were assessed based on knee X-ray and MRI findings. In cases of disagreement between the two evaluators, a senior radiology expert conducted a review and rendered the final diagnosis to ensure the objectivity and consistency of radiographic grading.

During arthroscopic examination or open replacement surgery under direct visualization, the attending surgeon assessed the status of the synovial tissue, including the degree of synovial hyperemia, proliferation, and vascularization, and observed the extent of structural damage to the cartilage surface and the presence of loose bodies. Synovial tissue samples were collected from multiple anatomical sites (e.g., the intercondylar fossa, suprapatellar pouch, and synovial plica regions) according to predefined criteria. All samples were immediately aliquoted under sterile conditions, snap-frozen in liquid nitrogen, and subsequently transferred to a −80 °C freezer for long-term storage prior to proteomic and metabolomic analyses.

### Data-independent acquisition (DIA) proteomics

During surgery, cartilage samples were collected under sterile conditions from multiple sites, including the femoral condyle and tibial plateau, from each participant. The samples were immediately snap-frozen in liquid nitrogen and transferred to a −80 °C freezer for storage until analysis. Tissues were ground into powder in liquid nitrogen and lysed in a buffer containing 8 M urea, 50 mM Tris-HCl (pH 8.0), and protease inhibitors. The mixture was sonicated on ice and centrifuged at 14,000 × g for 15 min at 4 °C, and the supernatant was collected for subsequent experiments. Protein concentration was determined using the BCA assay. Samples were reduced with 10 mM DTT at 56 °C for 30 min, followed by alkylation with 20 mM IAA in the dark at room temperature for 30 min. Filter-aided sample preparation (FASP) was performed using 10 kDa centrifugal filter units, and proteins were digested with trypsin (enzyme:protein ratio 1:50) at 37 °C overnight, followed by an additional 2 h digestion with a top-up of trypsin (1:100). The resulting peptides were desalted using C18 solid-phase extraction columns, vacuum-concentrated, and reconstituted in 0.1% formic acid aqueous solution. Equal aliquots from each sample were pooled to prepare a quality control (QC) sample for monitoring system stability.

Proteomic analysis was performed using liquid chromatography–tandem mass spectrometry based on DIA on a high-resolution Thermo Orbitrap Fusion Lumos mass spectrometer. Peptide separation was carried out on an Easy-nLC 1,200 system equipped with a nanoflow C18 reversed-phase column (25 cm × 75 μm, 2 μm particle size) at a flow rate of 300 nL/min. The gradient was linearly increased from 5% to 30% acetonitrile containing 0.1% formic acid over 90 min. Mass spectrometry parameters were set as follows: MS1 resolution 60,000 (at 200 m/z), scan range 350–1,550 m/z, AGC target 1.0e6, and maximum injection time 50 ms; MS2 resolution 30,000, normalized collision energy (NCE) 28–32, and DIA isolation windows were configured according to sample complexity (e.g., 24 windows of 25 m/z or a variable window strategy). A QC sample was injected after every 6–8 runs to monitor signal drift and batch effects. All raw mass spectrometry data were processed using DIA-NN software for peptide and protein identification and quantification. The Uniprot human reference proteome database was used for searching, and the false discovery rate (FDR) for both peptides and proteins was controlled at <1%.Proteins with a missing value rate exceeding 50% were removed. The remaining data were log2-transformed and normalized based on total peptide intensity. Random missing values were imputed using the K-nearest neighbor (KNN) algorithm or the minimum value substitution method. Differential expression analysis was performed using Student’s t-test, with significance defined as Fold Change ≥1.5 or ≤0.67 and Benjamini–Hochberg-adjusted p-value <0.05. Differentially expressed proteins were visualized using principal component analysis (PCA), heatmaps, and volcano plots. Functional enrichment analysis, including GO biological processes (BP), cellular components (CC), molecular functions (MF), and KEGG pathway enrichment, was conducted using the STRING database (https://cn.string-db.org/), with a significance threshold set at FDR <0.05. PPI analysis was performed using STRING (interaction score >0.7), followed by module clustering with MCODE and hub protein identification using CytoHubba in Cytoscape software. Candidate proteins were prioritized using a stepwise strategy. First, proteins meeting the predefined differential expression criteria were included. Second, their topological features in the PPI network, including degree ranking and network centrality, were evaluated. Third, GO/KEGG functional annotations were considered to determine whether these proteins were related to synovial inflammation, cytoskeletal remodeling, mitochondrial function, protein translation, or abnormal energy metabolism, which were the main biological processes of interest in this study. Finally, representative candidate molecules were selected for Western blot validation by taking into account previous literature, functional consistency with the metabolomic results, and experimental feasibility.

### Metabolomics

During surgery, tissue samples were collected from multiple sites of each participant under sterile conditions, immediately snap-frozen in liquid nitrogen, and stored at −80 °C. Tissue specimens were ground into powder in liquid nitrogen, and metabolites were extracted using ice-cold methanol/water (4:1, v/v) or methanol/acetonitrile solution. The mixture was vortexed, sonicated, and centrifuged at 14,000 × g for 15 min at 4 °C. The supernatant was collected, vacuum-dried at −20 °C, and reconstituted in an appropriate mobile phase. Lipids were extracted using the MTBE method to obtain the organic phase. Stable isotope-labeled internal standards were added during extraction to correct for quantitative errors. Pooled QC samples, prepared by mixing equal aliquots from each sample, were injected every 6–8 runs to monitor instrument stability. Analyses were performed using a UHPLC–MS/MS system (Thermo Q Exactive Plus). Polar metabolites were separated on a HILIC column, and lipids were separated on a C18 column at a flow rate of 0.3 mL/min. Data were acquired in both positive and negative ion modes. MS1 resolution was set at 70,000, with a scan range of 70–1,000 m/z. HCD collision energy ranged from 20 to 40 eV. Raw data were processed using XCMS or MS-DIAL for peak picking, alignment, and denoising. Features with a CV > 30% in QC samples were excluded. Data were normalized by total ion intensity or internal standard signals, and missing values were imputed using KNN or minimum value substitution. Metabolite annotation was based on accurate mass (≤5 ppm), MS/MS fragmentation spectra, and database matching (HMDB, METLIN, KEGG). Multivariate statistical analysis was performed using PCA and OPLS-DA. Differential metabolites were screened based on VIP >1 and p < 0.05 (FDR-corrected), and visualized using volcano plots and heatmaps. Pathway enrichment analysis was conducted using KEGG to identify pathways related to energy metabolism, amino acid metabolism, and inflammatory responses.

### Statistical analysis

All statistical analyses were performed using GraphPad Prism 9.0 (GraphPad Software, USA). Data are presented as mean ± standard deviation (SD). Comparisons between two groups were conducted using the independent samples Student’s t-test, and a *p*-value <0.05 was considered statistically significant. Statistical analyses were also performed using IBM SPSS version 29.0.

## Result

### Analysis of differential protein expression and functional enrichment in the synovial membrane of the knee joint

PCA analysis of proteomics data reveals the distribution of samples from the OA group and the control group within the principal component space (Supplementary Figure S1A). Each point represents an independent sample, with distinct colors indicating synovial membrane (IS) versus cartilage (DC) tissue, and different shapes differentiating the OA group from the normal control group. PCA of the synovial protein expression data showed a tendency toward separation between the OA and control groups. The model exhibited strong reproducibility and reliable grouping. The heat map further validated the consistency of protein changes between the groups (Supplementary Figure S1B). Additionally, the volcano plot illustrates the significant differences in protein expression between the normal and inflammatory groups (Figure 1A). Ultimately, 375 high-quality differentially expressed proteins were identified in the synovial proteome, with 202 proteins upregulated and 173 downregulated (Supplementary Figure S1A). To investigate the biological functions of differential proteins, we conducted GO enrichment analysis and KEGG enrichment analysis. GO enrichment analysis showed that the differentially expressed proteins in synovial tissue were mainly enriched in terms such as focal adhesion, lamellipodium, collagen-containing extracellular matrix, and cadherin binding, indicating enhanced cell adhesion, cell migration, and extracellular matrix remodeling. Notably, the significant enrichment of the extracellular exosome term suggested active intercellular communication, implying that OA synovium may be in a sustained inflammatory activation state associated with cell-to-cell signaling. KEGG enrichment analysis showed that the synovial differentially expressed proteins were mainly enriched in pathways including Ribosome, Spliceosome, Protein export, Nucleotide metabolism, Purine metabolism, and Pentose phosphate pathway. Among these, enrichment of Ribosome, Spliceosome, and Protein export suggested increased protein synthesis and post-transcriptional regulatory activity in synovial tissue, whereas enrichment of Purine metabolism, Nucleotide metabolism, and Pentose phosphate pathway indicated marked nucleotide metabolic remodeling. These findings suggest that OA synovium may exhibit a metabolically activated state characterized by nucleotide metabolic remodeling and increased protein synthesis, thereby providing a molecular basis for sustained inflammatory responses and tissue remodeling (Figures 1B,C). The degree table showed that POLR2C, COL1A1, EIF1AX, and TIAM1 had relatively high network connectivity (Supplementary Figure S1B). We further found that TIAM1 was located within the interaction network and formed interactions with multiple proteins (Figure 1D).

**FIGURE 1:**
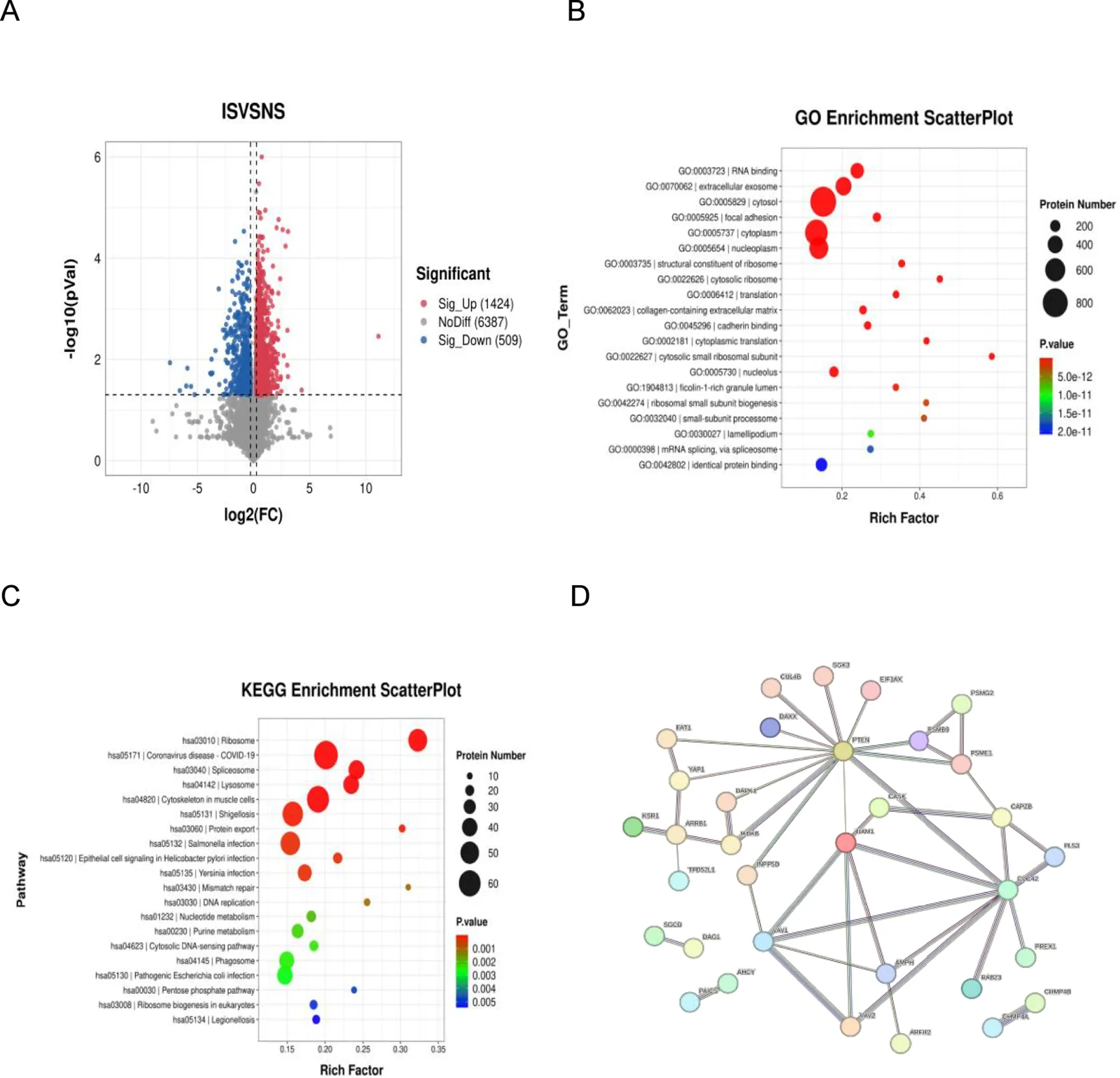
Proteomic profiling of synovial tissue in KOA. **(A)** Volcano plot showing DEPs between OA (IS) and normal synovium (NS) (|Fold Change| ≥ 1.5, adjusted *p* <0.05). Red: upregulated; blue: downregulated. **(B)** GO enrichment of synovial DEPs. The x-axis shows rich factor; bubble size indicates protein count; color denotes significance (*p*-value). **(C)** KEGG pathway enrichment. Pathways are primarily related to inflammation, cell adhesion, and metabolism. **(D)** PPI network of synovial DEPs constructed using STRING. Nodes represent proteins, and edges represent known or predicted protein–protein interactions.

### Analysis of differential protein expression and functional enrichment in knee joint cartilage

PCA of the cartilage protein expression data showed a tendency toward separation between the OA and control groups (Supplementary Figure S2A). The heat map corroborated the consistency of protein alterations across the groups (Supplementary Figure S2B). Volcano plots further depicted the notable differences in protein expression between the normal and inflammatory groups (Figure 2A). Ultimately, a total of 57 high-quality differentially expressed proteins were identified within the chondroproteome, comprising 18 up-regulated and 39 down-regulated proteins (Supplementary Table S2A). To investigate the biological functions of these differential proteins, both GO enrichment analysis and KEGG enrichment analysis were performed. GO enrichment analysis showed that the differentially expressed proteins in cartilage were mainly enriched in terms such as focal adhesion, lamellipodium,collagen-containing extracellular matrix,and translation repressor activity. These results suggest that OA cartilage may exhibit alterations in extracellular vesicle-related material exchange, coagulation/fibrinolysis-related processes, cytoskeletal or contractile structures, and post-transcriptional regulation. Enrichment of cellular component terms such as cytosol and extracellular exosome further suggested alterations in intracellular and extracellular material exchange and the metabolic regulatory environment. KEGG enrichment analysis further showed that cartilage differentially expressed proteins were mainly enriched in energy and basal metabolic pathways, including Glycolysis/Gluconeogenesis, Carbon metabolism, Citrate cycle (TCA cycle),Pyrimidine metabolism, and Metabolic pathways. Taken together, these results suggest that OA cartilage may undergo metabolic remodeling involving glycolysis, central carbon metabolism, the TCA cycle, nucleotide-related metabolism, and amino acid biosynthesis. Combined with the downregulation of MRPS7 and UCK1 and the metabolomic decrease in energy metabolism-related intermediates, these findings support the presence of restricted energy metabolism, impaired basal metabolic capacity, and cellular stress-related changes in OA cartilage. (Figures 2B,C). The degree table showed that ACTN3, MRPS7, MDH2, and PGAM2 had relatively high network connectivity (Supplementary Table S2A). We further found that MRPS7 was located within the interaction network and formed interactions with multiple proteins (Figure 2D).

**FIGURE 2:**
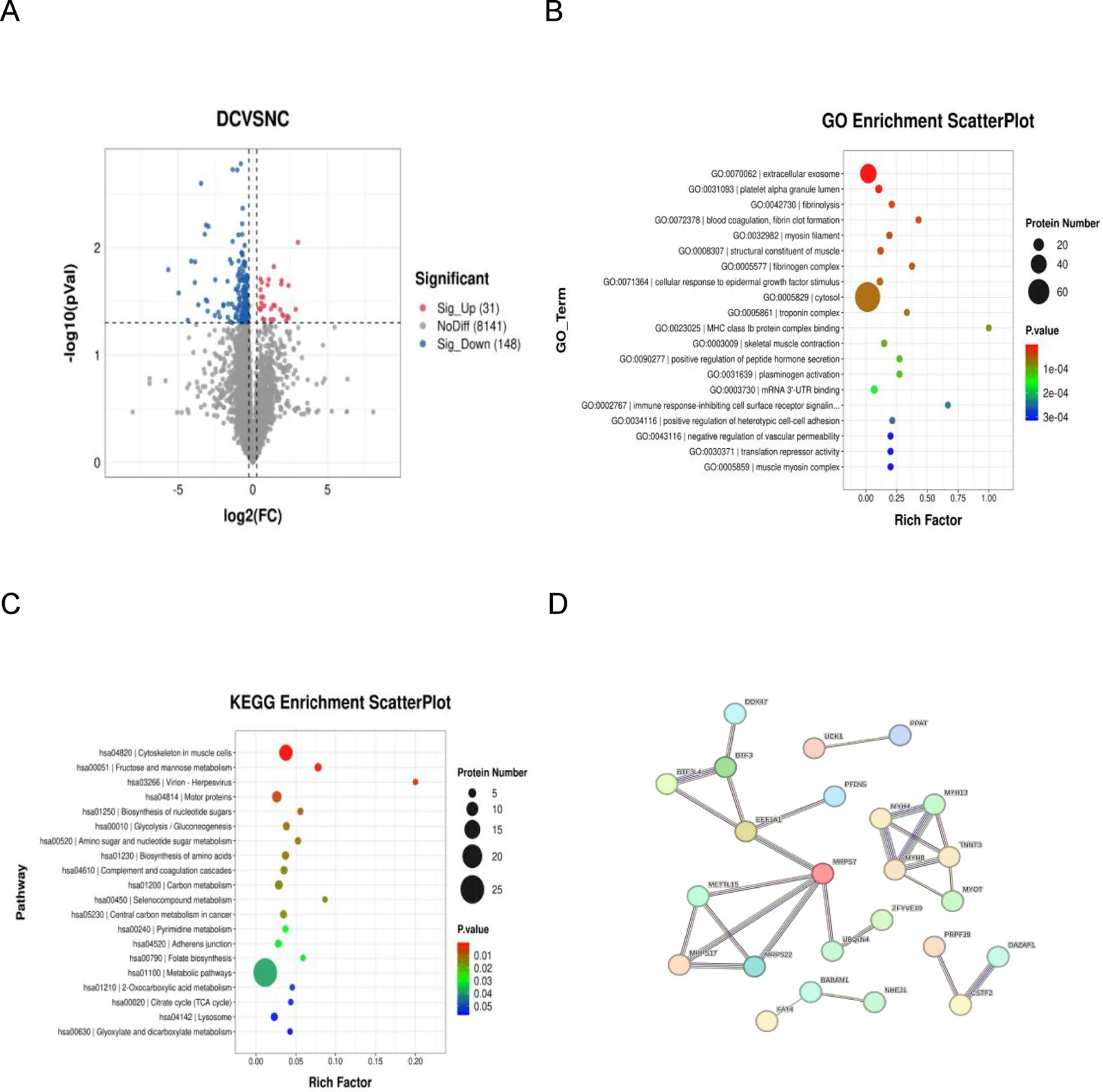
Proteomic profiling of cartilage tissue in KOA. **(A)** Volcano plot showing DEPs between OA (DC) and normal cartilage (NC) (|Fold Change| ≥ 1.5, adjusted *p* <0.05). Red: upregulated; blue: downregulated. **(B)** GO enrichment of cartilage DEPs. The x-axis represents rich factor; bubble size indicates protein count; color denotes significance (*p*-value). **(C)** KEGG pathway enrichment. Enriched pathways are primarily associated with energy metabolism, the citrate cycle (TCA cycle), and carbon metabolism. **(D)** PPI network of cartilage DEPs constructed using STRING. Nodes represent proteins, and edges represent known or predicted protein–protein interactions.

### Western blot validation

To verify the reliability of the DIA-based proteomic findings, we performed Western blot analysis on independent synovial and cartilage samples, focusing on the selected hub proteins in the above PPI networks (Figure 3). In synovial tissue, compared with the control group, the protein level of TIAM1 was significantly increased in the osteoarthritis (OA) group, which was consistent with the proteomic data. In cartilage tissue, compared with the control group, MRPS7 expression was significantly decreased in the OA group, which was consistent with the proteomic results and further confirmed the view that mitochondrial translation capacity is impaired in OA cartilage. These validation results confirmed the robustness of the proteomic analysis and strengthened the biological relevance of the identified molecular changes. Notably, in the DIA proteomic data, Rac1 did not reach the predefined differential expression threshold (fold change ≥1.5, p < 0.05), which may be related to the fact that its activity is mainly regulated by post-translational mechanisms such as GDP/GTP exchange, while changes in total protein levels are relatively small. However, given the clear biological function of Rac1 as a key downstream effector of TIAM1 and its core role in amplifying inflammatory signaling, we still performed independent validation of its total protein level. Western blot results showed that, compared with the control group, Rac1 expression was significantly increased in OA synovial tissue, which was highly consistent with the upregulation trend of its upstream regulator TIAM1.

**FIGURE 3:**
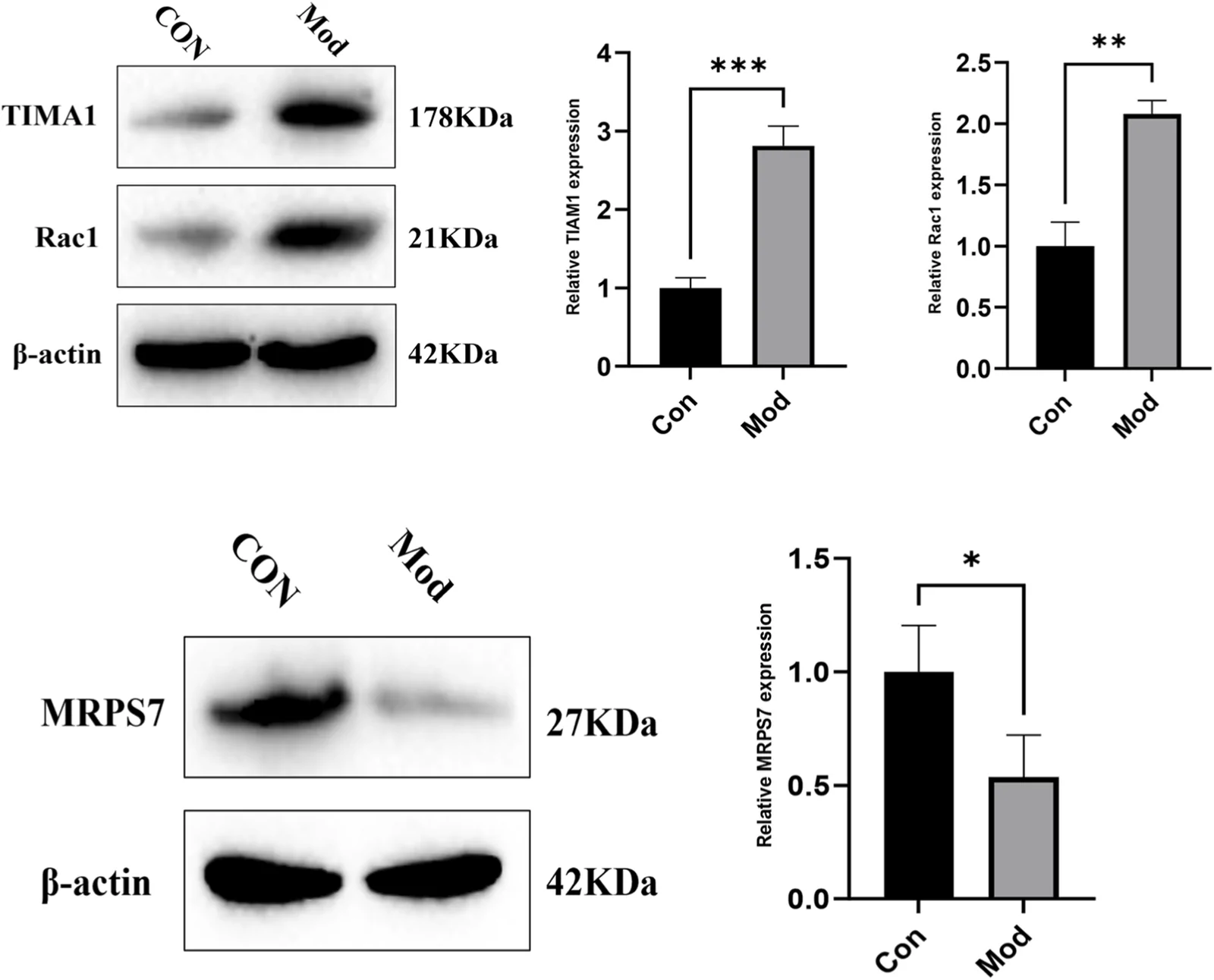
Western blot validation of key differentially expressed proteins. (Upper panel) Representative Western blot images and quantitative analysis of TIAM1 and Rac1 protein expression in synovial tissues. Compared with the control group (Con), the osteoarthritis group (Mod) showed significantly elevated expression of TIAM1 (178 kDa) and Rac1 (21 kDa). β-actin (42 kDa) served as the loading control. (Lower panel) Representative Western blot images and quantitative analysis of MRPS7 protein expression in cartilage tissues. Compared with the control group, MRPS7 (27 kDa) expression was significantly decreased in the OA group. β-actin (42 kDa) was used as the loading control. Data are presented as mean ± SD. **p < 0.01, ***p < 0.001 compared with control group.

### Metabolomic profiling analysis of articular cartilage and synovial tissue in the knee joint

Metabolomics analysis was subsequently performed to investigate the metabolic alterations in the synovial membrane and cartilage. This study implemented stringent quality control measures on the original LC-MS/MS data. QC samples were established, and Pearson correlation analysis was employed to assess detection repeatability. The results indicated that the correlation coefficients among QC samples were all ≥0.988, demonstrating the detection platform’s stability and consistency (Supplementary Figure S4A). Additionally, the CV for each metabolic characteristic was calculated, with a CV threshold of <30% used as the criterion to exclude low-stability signals exhibiting significant technical fluctuations. Building on this foundation, PLS-DA was utilized to evaluate model reliability. The 200-permutation test revealed that the intercept of Q^2^ was <0, R^2^ exceeded Q^2^, and the regression line intersected the negative region of the Y-axis, indicating that the model was not overfitted and that the observed differences between groups were both real and reliable (Supplementary Figures S4B, C). ROC analysis indicated that the top-ranked differential metabolites identified in the synovial membrane comparison exhibited strong discriminatory power. The AUC value for individual metabolites ranged from 0.91 to 0.94, while the AUC for the integrated logistic regression models of multiple metabolites reached 0.958 (95% CI: (0.91–1.00)), highlighting their potential as biomarkers for distinguishing OA from control synovial samples (Supplementary Figures S4D, E). The heat map further depicts the relative abundance of metabolites (Supplementary Figures S4F, G), and the volcano diagrams illustrate the differences in metabolite levels (Figures 4A,B). In the analysis of differentially abundant metabolites, after removing redundant peaks, a total of 125 differentially abundant metabolites were identified in synovial tissue, with 75 upregulated and 50 down-regulated. Additionally, 47 differential metabolites were identified in cartilage tissue, comprising 22 upregulated and 25 downregulated. KEGG analysis indicated that the differential metabolites in the synovial membrane were predominantly enriched in central carbon metabolism and various pathways associated with basic energy metabolism. This enrichment encompasses critical metabolic processes such as glycolysis and the tricarboxylic acid cycle (Figure 4C). Notably, several intermediates of energy metabolism exhibited a decline in worn cartilage, reflecting significant alterations in the energy metabolism levels and metabolic balance of chondrocytes under pathological conditions (Figure 4D). Additionally, gene set enrichment analysis (GSEA) revealed that the “arachidonic acid metabolism” pathway was significantly enriched in synovial tissue (NES = 2.22, FDR = 0) (Figure 4E). In contrast, pathways related to “central carbon metabolism” also demonstrated a trend of enrichment in cartilage tissue (NES = 1.80, FDR = 0.07) (Figure 4F). These findings suggest that the two tissues exhibit distinct metabolic changes characterized by enhanced inflammation-related lipid metabolism and suppressed energy metabolism under disease conditions.

**FIGURE 4:**
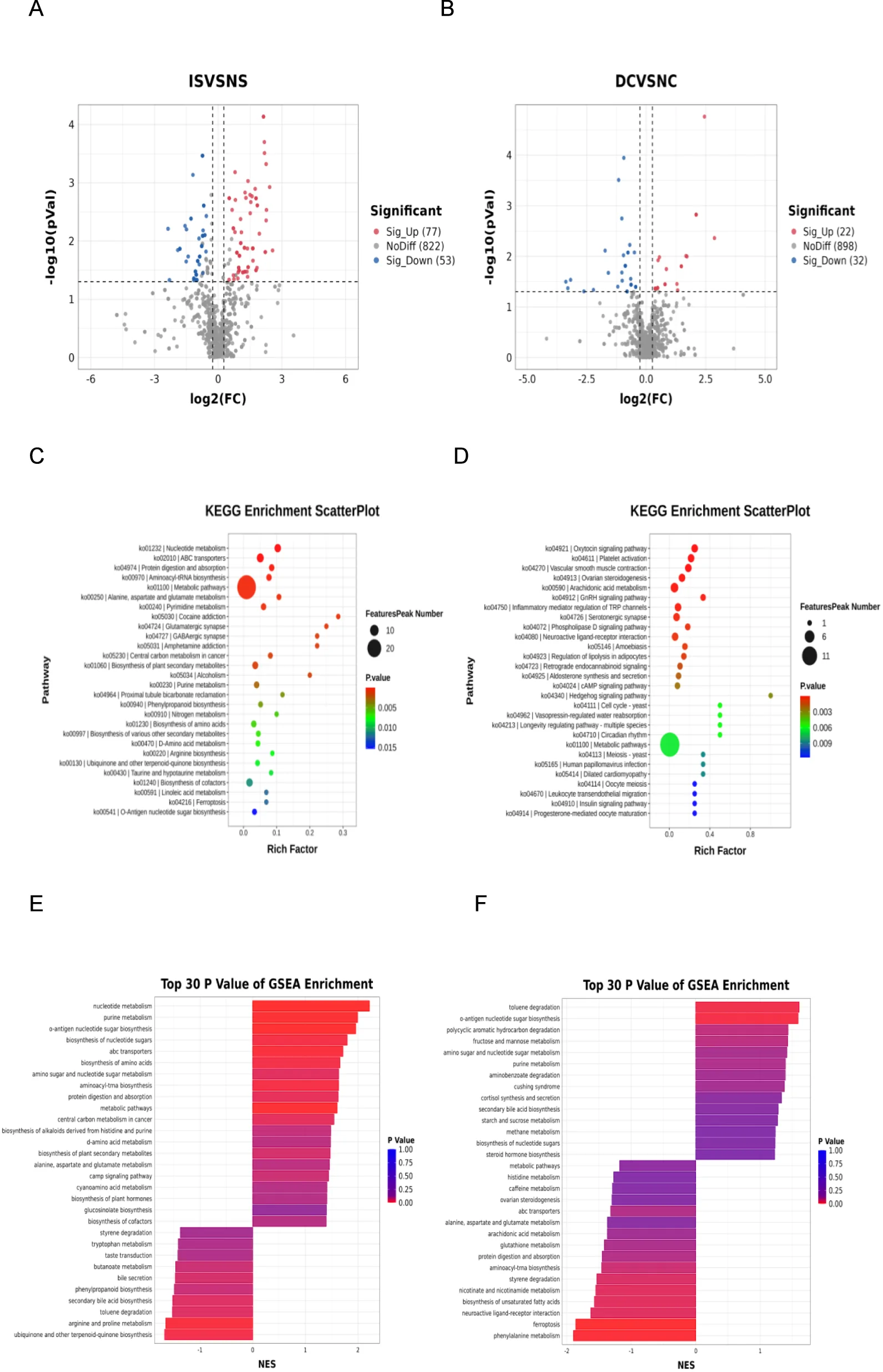
Metabolomic profiling of synovial and cartilage tissues in KOA. **(A,B)** Volcano plots showing differential metabolites in synovium **(A)** and cartilage **(B)** between OA and control groups. Each point represents a metabolite feature. Red: significantly upregulated in OA; blue: significantly downregulated; gray: not significant (VIP >1, *p* <0.05). **(C,D)** KEGG pathway enrichment of differential metabolites in synovium **(C)** and cartilage **(D)**. The y-axis lists enriched pathways; bubble size indicates metabolite count; color denotes significance (*p*-value). **(E,F)** GSEA results showing top 30 enriched pathways in synovium **(E)** and cartilage **(F)**. The x-axis represents normalized enrichment score (NES); color indicates significance.

### Integrated protein–metabolite analysis of knee synovium

To further clarify the coupling relationship between protein alterations and metabolic remodeling in OA synovium, we performed joint KEGG enrichment analysis and protein–metabolite correlation analysis of the differentially expressed proteins and differential metabolites in synovial tissue (Figures 5A,B). The joint pathway analysis showed that synovial differentially expressed proteins and differential metabolites were commonly focused on nucleotide metabolism, purine/pyrimidine metabolism, carbon metabolism, amino sugar and nucleotide sugar metabolism, as well as pathways related to oxidative stress/lipid peroxidation. These findings suggest that OA synovium not only exhibits abnormal expression of inflammation-related proteins, but is also accompanied by coordinated remodeling of basal metabolism and inflammation-related metabolic networks. Further correlation heatmap analysis showed that proteins related to small GTPase regulation, signal transduction, and regulation of cellular behavior, including TIAM1, PREX1, RABGEF2, AHCY, ARRB1, and TPD52L1, formed consistent correlation modules with multiple differential metabolites. Among them, several amino acid/dipeptide, nucleotide-related, and redox-related metabolites showed coordinated changing trends with these proteins, supporting the parallel occurrence of synovial inflammatory signaling activation and metabolic reprogramming. Combined with the network centrality of TIAM1 in the proteomic analysis, the upregulation of Rac1 protein in Western blot analysis, and the increase in arachidonic acid metabolism-related lipid mediators in the metabolomic analysis, these results suggest that OA synovium not only shows abnormal expression of inflammation-related proteins, but is also accompanied by extensive remodeling of networks related to nucleotide metabolism, amino acid metabolism, and carbohydrate metabolism.

**FIGURE 5:**
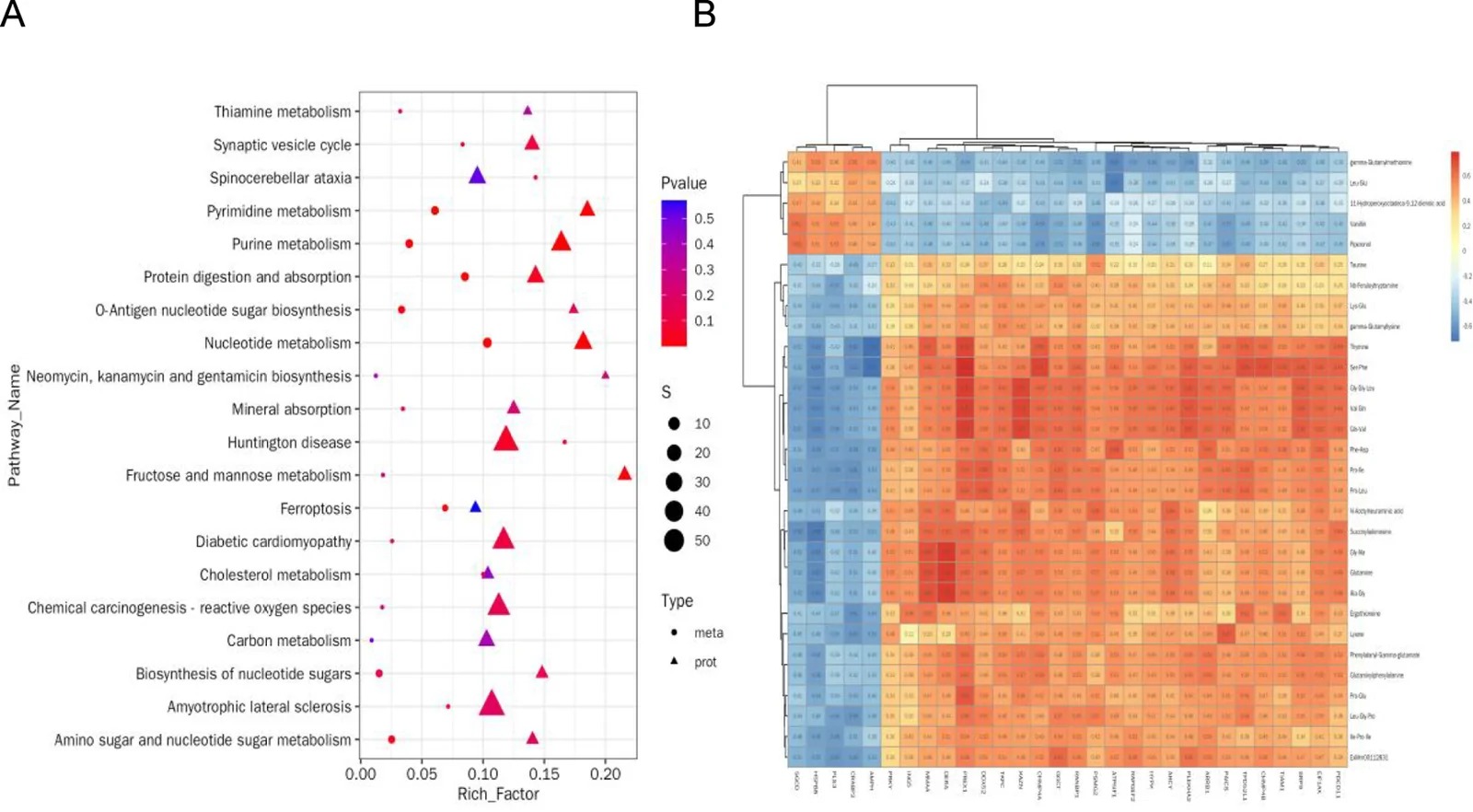
**(A)** Shows the joint KEGG enrichment scatter plot of the main pathways shared by differentially expressed proteins and differential metabolites in OA synovial tissue. The x-axis represents the enrichment factor (Rich factor), and the y-axis represents the significantly enriched KEGG pathway names. Bubble size indicates the number of differential molecules included in each pathway, and color represents the enrichment significance level (p value). Circles indicate differential metabolites (meta), and triangles indicate differentially expressed proteins (prot). **(B)** shows the protein–metabolite correlation heatmap displaying the correlations between synovial differentially expressed proteins and differential metabolites. The x-axis represents differentially expressed proteins, and the y-axis represents differential metabolites. The color of each block indicates the correlation coefficient, with red indicating positive correlations and blue indicating negative correlations. Hierarchical clustering based on correlation patterns is also shown in the figure.

### Integrated metabolite–protein analysis of knee cartilage

To reveal the relationship between protein abnormalities and restricted metabolic function in OA cartilage, we further performed joint KEGG enrichment analysis and protein–metabolite correlation analysis of cartilage differentially expressed proteins and differential metabolites (Figures 6A,B). The joint pathway analysis showed that cartilage differentially expressed proteins and differential metabolites were mainly focused on basic metabolic pathways, including metabolic pathways, purine metabolism, cysteine and methionine metabolism, alanine/aspartate/glutamate metabolism, and fructose and mannose metabolism. At the protein level, stress-related processes such as MAPK signaling, calcium signaling, and necroptosis were also involved, suggesting that metabolic restriction and cellular stress coexist in OA cartilage. Protein–metabolite correlation analysis further showed that cartilage differentially expressed proteins formed clear correlation modules with lipids, phospholipids, purine metabolism-related molecules, and some energy metabolism-related metabolites, supporting coordinated changes in membrane lipid homeostasis, nucleotide metabolism, and energy metabolism in cartilage. Combined with the above proteomic and metabolomic results, OA cartilage showed a molecular profile characterized by restricted energy metabolism, reduced mitochondria-related function, and structural/stress remodeling. Taken together, the joint KEGG enrichment analysis and protein–metabolite correlation analysis indicate broad associations between protein expression abnormalities and metabolite alterations in OA cartilage, mainly involving purine metabolism, amino acid metabolism, lipid metabolism, and cell signaling-related processes.

**FIGURE 6:**
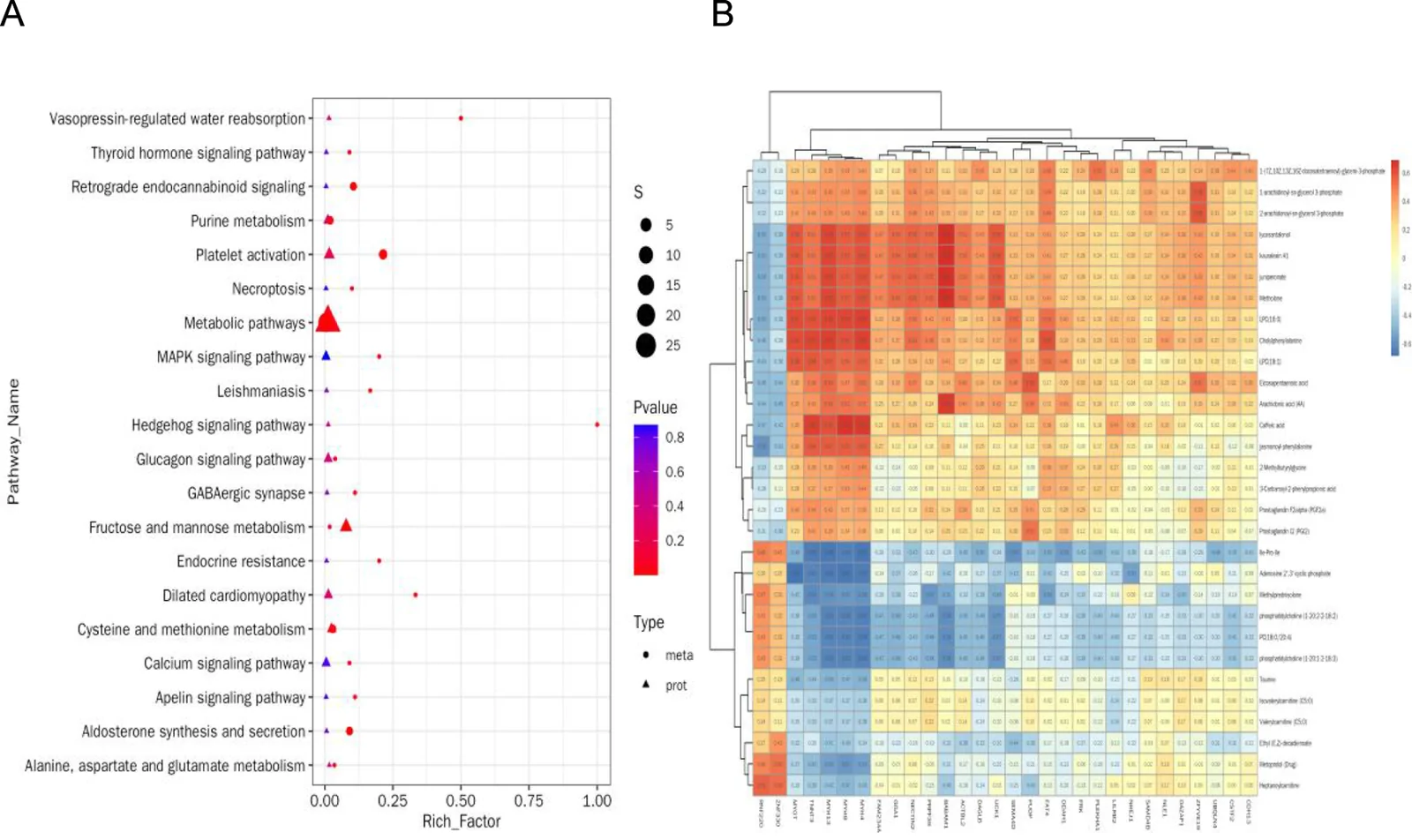
**(A)** shows the joint KEGG enrichment scatter plot of the main pathways shared by differentially expressed proteins and differential metabolites in OA cartilage tissue. The x-axis represents the enrichment factor (Rich factor), and the y-axis represents the significantly enriched KEGG pathway names. Bubble size indicates the number of differential molecules included in each pathway, and color represents the enrichment significance level (p value). Circles indicate differential metabolites (meta), and triangles indicate differentially expressed proteins (prot). **(B)** shows the protein–metabolite correlation heatmap displaying the correlations between cartilage differentially expressed proteins and differential metabolites. The x-axis represents differentially expressed proteins, and the y-axis represents differential metabolites. The color of each block indicates the correlation coefficient, with red indicating positive correlations and blue indicating negative correlations. Hierarchical clustering based on correlation patterns is also shown in the figure.

### Cross-tissue integrated analysis of synovium and cartilage

After completing the protein–metabolite integrated analyses within synovial and cartilage tissues separately, we further performed a cross-tissue comparison of molecular alterations in these two tissues (Figure 7). The results showed that OA synovium and cartilage did not present as isolated molecular abnormalities, but instead displayed functionally complementary alteration patterns involving inflammatory signaling, lipid metabolism, nucleotide metabolism, energy metabolism, and cellular stress. In synovial tissue, the differentially expressed proteins were mainly involved in inflammatory signal transduction, cytoskeletal remodeling, and cell migration-related processes. Representative molecules included TIAM1, Rac1-related regulatory proteins, PREX1, RABGEF2, and ARRB1. Meanwhile, the synovial differential metabolites and joint enrichment results suggested remodeling of pathways such as nucleotide metabolism, purine metabolism, pyrimidine metabolism, fructose metabolism, and carbon metabolism, accompanied by increased inflammatory lipid mediators, including PGE2, TXB2, 12-HETE, and arachidonic acid. These results suggest that OA synovial tissue is in a state of inflammatory signaling activation and enhanced metabolic output. In contrast to the inflammatory–metabolic activation features of synovium, OA cartilage mainly showed restricted mitochondrial function, translational regulation, and basal energy metabolism. Proteomic results in cartilage showed alterations in molecules related to mitochondrial translation, nucleotide metabolism, and protein synthesis, including MRPS7, UCK1, and EIF5A. Cartilage metabolomics and protein–metabolite joint enrichment analysis further showed that processes including metabolic pathways, purine metabolism, fructose and mannose metabolism, MAPK signaling pathway, calcium signaling pathway, necroptosis, and ECM–receptor interaction were affected, accompanied by decreased or abnormal levels of energy metabolism-related indicators such as citrate, pyruvate, lactate, and cAMP. These findings suggest that OA cartilage may be characterized by insufficient energy supply, impaired mitochondrial function, increased cellular stress, and disrupted matrix homeostasis. Taken together, the multi-omics results from the two tissues indicate that OA synovium is mainly characterized by inflammatory signaling, lipid mediator release, and metabolic remodeling, whereas cartilage is mainly characterized by impaired mitochondrial/translational function, restricted central carbon metabolism, and matrix damage. At the functional level, these changes form a cross-tissue alteration pattern in which “synovial inflammatory–lipid metabolic activation” coexists with “cartilage energy metabolic restriction.” These findings suggest that OA is not merely a disease of cartilage degeneration, but rather a whole-joint disorder involving synovial inflammatory–metabolic activation and cartilage metabolic functional decline.

**FIGURE 7:**
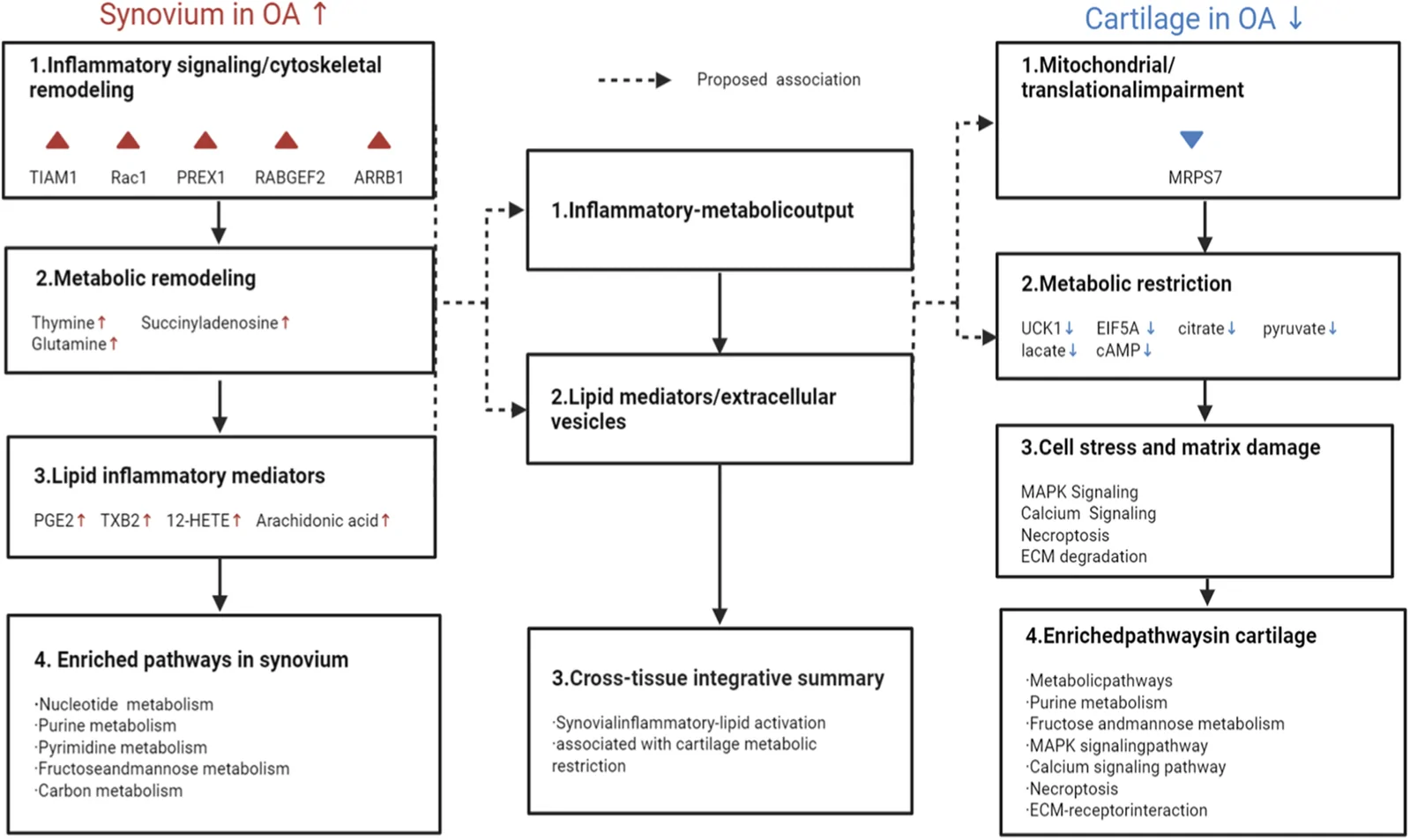
Cross-tissue multi-omics integrative model of synovium and cartilage. Based on differentially expressed proteins, differential metabolites, joint KEGG enrichment analysis, and protein–metabolite correlation analysis, this figure summarizes the major molecular alterations in OA synovial and cartilage tissues. OA synovium is mainly characterized by enhanced inflammatory signaling/cytoskeletal remodeling, remodeling of nucleotide and carbohydrate metabolism, and increased inflammatory lipid mediators, including PGE2, TXB2, 12-HETE, and arachidonic acid. In contrast, OA cartilage is mainly characterized by MRPS7-related impairment of mitochondrial/translational function, decreased UCK1/EIF5A and central carbon metabolism-related molecules, increased cellular stress, and ECM degradation. The middle section illustrates synovial inflammatory–metabolic output, lipid mediators/extracellular vesicles, and their potential cross-tissue associations with restricted cartilage metabolism. Red arrows indicate molecules/processes that are upregulated or enhanced in OA synovium, whereas blue arrows indicate molecules/processes that are downregulated or restricted in OA cartilage. Solid arrows indicate functional relationships within each tissue, whereas dashed arrows indicate potential cross-tissue associations rather than proven causal relationships.

## Discussion

In previous studies, chondrocytes have been identified as the primary regulators of cartilage metabolism (Rahman et al., 2023). During the progression of KOA, chondrocytes in articular cartilage experience a range of mechanical stimuli. These mechanical signals traverse a complex network that includes the ECM, pericellular matrix (PCM), cytoskeleton, and chondrocyte nuclei. In response to these stimuli, chondrocytes modify gene expression and metabolic activity (Chen et al., 2013). The principal factor driving chondrocyte death is strain amplification, wherein the maximum principal strain and shear strain in the ECM, PCM, and intracellular environment exceed a critical threshold. Rapid shock loading, in contrast to slow loading, exacerbates strain within the ECM, PCM, and intracellular compartments. This amplification significantly diminishes the viability of articular chondrocytes and compromises the stiffness of the cartilage matrix (Argote et al., 2019). Current research has redefined OA as a “whole joint disease,” wherein each compartment—including articular cartilage, synovial membrane, subchondral bone, subpatellar fat pad, ligaments, and meniscus—contributes to its progression. Notably, the synovial membrane has transitioned from a supportive role to a central one due to its critical function in initiating an immune response in OA (Mathiessen and Conaghan, 2017). Synovitis serves as a primary driver of OA progression and pain. An imbalance in chondrocyte homeostasis results in the degradation of the ECM, leading to the release of DAMPs and PAMPs. These molecules bind to pattern recognition receptors (PRRs) on the surface of synovial innate immune cells, activating these immune cells and initiating inflammatory responses. This process exacerbates joint tissue damage through the release of inflammatory mediators, thereby creating a vicious cycle (Panichi et al., 2024). However, most contemporary studies depend on single-dimensional omics analyses and do not adequately integrate multi-level data, including proteomics, metabolomics, and epigenomics. This limitation disrupts the “molecular-function-pathology” association. Furthermore, current omics investigations frequently focus on a single tissue, such as analyzing chondrocytes or subchondral osteocytes in isolation, thereby overlooking the interactions among cells from different tissues,and further provided an integrated interpretation of the molecular abnormalities in the two tissues through protein–metabolite joint analysis.

In this study, we performed a metabolomics analysis using LC-MS/MS. We initially examined the synoviomics of KOA and identified 125 significantly altered metabolites in the synovium of KOA patients, comprising 75 upregulated and 50 downregulated metabolites, with lipid metabolism being the most prominent. Notably, inflammation-related metabolism, particularly the arachidonic acid metabolic pathway, was significantly enhanced. A range of typical inflammatory lipid mediators, including PGE2, TXB2, and 12-HETE, were significantly upregulated in the synovial membrane of OA. These findings suggest that the synovial tissue demonstrates persistent inflammation-related metabolic output characteristics under disease conditions. PGE_2_ is a principal member of the prostaglandin family, synthesized from arachidonic acid via COX and PGES. It is extensively involved in both physiological and pathological processes, including inflammatory responses, pain transmission, and tissue repair, and it plays a crucial regulatory role in the onset and progression of OA (Park et al., 2006; Chwastek et al., 2025). Similarly, 12-HETE is a significant lipid metabolite produced by 12-LOX from arachidonic acid and belongs to the HETE family. This metabolite is actively engaged in various physiological and pathological processes, such as inflammation regulation, cell proliferation, oxidative stress, and tissue remodeling, and it is integral to the pathophysiology of numerous diseases (Zhang XJ. et al., 2018). Previous studies have demonstrated that both molecules are crucial regulatory components of the Rac1 signaling pathway (Wen et al., 2000; Zhang et al., 2018a). The involvement of Rac1 in arthritis-related inflammatory responses has been extensively investigated. Its aberrant activation contributes not only to the migration, adhesion, and cytoskeletal remodeling of synovial cells but also enhances the downstream effects of various inflammatory signaling pathways, including the promotion of inflammatory factors and chemokine production. The existing literature indicates that Rac1 signaling can modulate the release of key inflammatory mediators, such as IL-1β and TNF-α, which play a fundamental role in shaping the local inflammatory microenvironment of the joints (Liu X. et al., 2024; Jiang et al., 2017; Onuora, 2014; Zhu et al., 2015).

We subsequently conducted a detailed analysis of the protein profiles in the synovial membrane and cartilage of patients with KOA and in normal human subjects using DIA proteomics. Our analysis revealed 375 proteins with significant alterations in the synovium, comprising 202 upregulated and 173 downregulated proteins. In cartilage, we identified 57 proteins with significant changes in biological functions, including 18 that were upregulated and 39 that were downregulated. The results of synovial proteomics indicate that the differential proteins in the synovial membrane of OA are predominantly enriched in biological processes associated with inflammatory response, immune response regulation, protein translation, and cell adhesion. Concurrently, at the cellular composition level, these proteins are primarily localized in structures such as the cytoplasm, nucleus, and extracellular vesicles. This localization suggests that the synovial membrane is in a highly active state of inflammation and signal communication in the context of OA. These findings align closely with previous studies that identify the synovium as a critical tissue serving as an “amplifier” of the OA inflammatory response and a source of pain (Barnett, 2018). Further integrated synovial protein–metabolite analysis showed partial overlap between differentially expressed proteins and differential metabolites in nucleotide metabolism, purine/pyrimidine metabolism, carbohydrate metabolism, and oxidative stress-related pathways. This suggests that inflammatory signaling activation in OA synovium does not occur in isolation, but is accompanied by metabolic network remodeling and enhanced inflammatory mediator output. These findings provide multi-omics support for the role of synovium as an inflammatory–metabolically activated tissue in OA.

Notably, along with the enhanced metabolism of the arachidonic acid pathway, we detected a significant upregulation of TIAM1 in OA synovium. This protein was located within the major protein–protein interaction network of synovial tissue and showed relatively high network connectivity. Some highly connected proteins, such as COL1A1, have been widely reported to participate in pathological processes including OA cartilage degeneration, synovial fibrosis, and joint tissue remodeling, and their roles in OA have been relatively well studied. In contrast, to date, few studies have directly linked TIAM1 to KOA. Previous studies have confirmed that TIAM1 is a specific guanine nucleotide exchange factor (GEF) for Rac1, which can promote Rac1 activation and participate in Rac1-mediated signal regulation in various biological processes (Song et al., 2023; Xu et al., 2010). Combined with the significant upregulation of TIAM1 in OA synovial tissue observed in this study and its presence in the main PPI network, we considered that TIAM1 may participate in KOA-related pathological processes by regulating Rac1, and therefore selected it as a candidate protein for further validation. In addition to TIAM1, VAV2 is also a key upstream molecule regulating Rac1 activity (Han et al., 2025). Interestingly, this study found that VAV2 was also significantly upregulated in OA synovium and was likewise located within the synovial protein interaction network. STRING interaction analysis showed a high-confidence interaction between TIAM1 and VAV2 (interaction score = 873). Therefore, their simultaneous upregulation further supports the potential involvement of the Rac1-related signaling network in abnormal activation of OA synovium. This finding provides new molecular clues for understanding the abnormal activation state of OA synovium.

Given that synovium had already exhibited an inflammatory invasive phenotype, we speculated that cartilage may undergo dual pressure on synthesis and energy metabolism, and this hypothesis was supported by cartilage metabolomics. In the cartilage metabolomic analysis, we detected 47 significantly altered differential metabolites, including 22 upregulated and 25 downregulated metabolites. Enrichment analysis revealed a metabolic remodeling pattern characterized mainly by suppression of energy metabolism and basal metabolic pathways in cartilage. Specifically, the overall levels of several central carbon metabolism-related metabolites were decreased, including key metabolic intermediates such as lactate, pyruvate, and citrate. These findings suggest reduced metabolic flux in energy-supplying pathways such as glycolysis and the tricarboxylic acid cycle in OA chondrocytes. Restricted energy metabolism and mitochondrial dysfunction have been repeatedly reported as important molecular bases during cartilage degeneration in KOA, and our metabolomic results further support this view at the small-molecule level.

Subsequently, we identified 57 proteins with significant biological changes in cartilage, including 18 upregulated and 39 downregulated proteins. In this study, both MRPS7 and UCK1 were found to be significantly downregulated in cartilage tissues from patients with KOA. MRPS7, as a mitochondrial ribosomal protein, participates in the translation of mitochondrially encoded proteins and plays an important role in maintaining mitochondrial function and cellular energy metabolic homeostasis (Connolly and Duley, 1999). Since mitochondrial dysfunction and impaired energy metabolism are among the important molecular bases of cartilage degeneration in KOA, the downregulation of MRPS7 reflects suppression of the mitochondrial translation system. At the same time, MRPS7 was also among the core interacting nodes in the PPI network and showed relatively high network connectivity, suggesting that it may participate in the regulation of molecular networks related to metabolic abnormalities in OA cartilage. Therefore, it was also selected as a candidate protein for further validation. UCK1, as a key enzyme in the pyrimidine nucleoside salvage synthesis pathway, participates in cellular nucleotide metabolism and maintains basic biological processes such as RNA synthesis and protein translation (Menezes et al., 2015). Chondrocytes generally show reduced synthetic capacity and restricted metabolic activity during the progression of KOA. In this study, the downregulation of UCK1 was consistent with the overall decreasing trend of central carbon metabolism-related metabolites observed in cartilage metabolomics, suggesting that OA chondrocytes may have simultaneous impairment of basal metabolism and synthetic capacity. The simultaneous decrease of these two proteins indicates that OA chondrocytes have abnormalities in two fundamental processes: nucleotide supply and energy production. Further integrated cartilage protein–metabolite analysis showed partial overlap between differentially expressed proteins and differential metabolites in basal metabolism, purine metabolism, carbohydrate metabolism, amino acid metabolism, and cellular stress-related pathways. This suggests that abnormal protein expression in OA cartilage does not occur in isolation, but is accompanied by metabolic abnormalities, leading to restricted energy metabolism, reduced mitochondrial function, and disruption of matrix homeostasis. These findings provide multi-omics support for the pathological state in which OA chondrocytes exhibit both metabolic functional decline and stress-related injury.

From the perspective of cross-tissue integration, this study found that OA synovium and cartilage did not exhibit isolated molecular abnormalities, but instead showed corresponding alteration patterns in inflammatory signaling, lipid metabolism, energy metabolism, and cellular stress. On the synovial side, the main features were enhanced inflammatory signaling, cytoskeletal remodeling, nucleotide/carbohydrate metabolic remodeling, and increased arachidonic acid metabolism-related lipid mediators, whereas the cartilage side was mainly characterized by impaired mitochondrial translation, restricted central carbon metabolism, increased cellular stress, and disruption of matrix homeostasis. Based on these findings, this study proposes a hypothetical cross-tissue imbalance model of “synovial invasive-like activation–enhanced inflammatory signaling output–restricted cartilage energy metabolism.” In synovium, enrichment of the arachidonic acid pathway, together with the upregulation of TIAM1 and VAV2, suggests enhanced Rac1-related inflammatory activation signaling, accompanied by significant increases in inflammation-related lipid metabolites such as PGE2 and 12-HETE, reflecting enhanced inflammatory–metabolic output of the synovium. In contrast, the downregulation of UCK1 and MRPS7 in cartilage showed consistent changes with the decrease of central carbon metabolism-related metabolites such as citrate and lactate, suggesting restricted energy supply and anabolic capacity in chondrocytes. Therefore, we speculate that the enhanced arachidonic acid metabolism and activation of its Rac1-related signaling axis in OA synovium may indirectly affect the metabolic homeostasis of chondrocytes by altering the inflammatory and metabolic state of the joint microenvironment, thereby aggravating impaired cartilage energy metabolism and functional deterioration (Figure 8).

**FIGURE 8:**
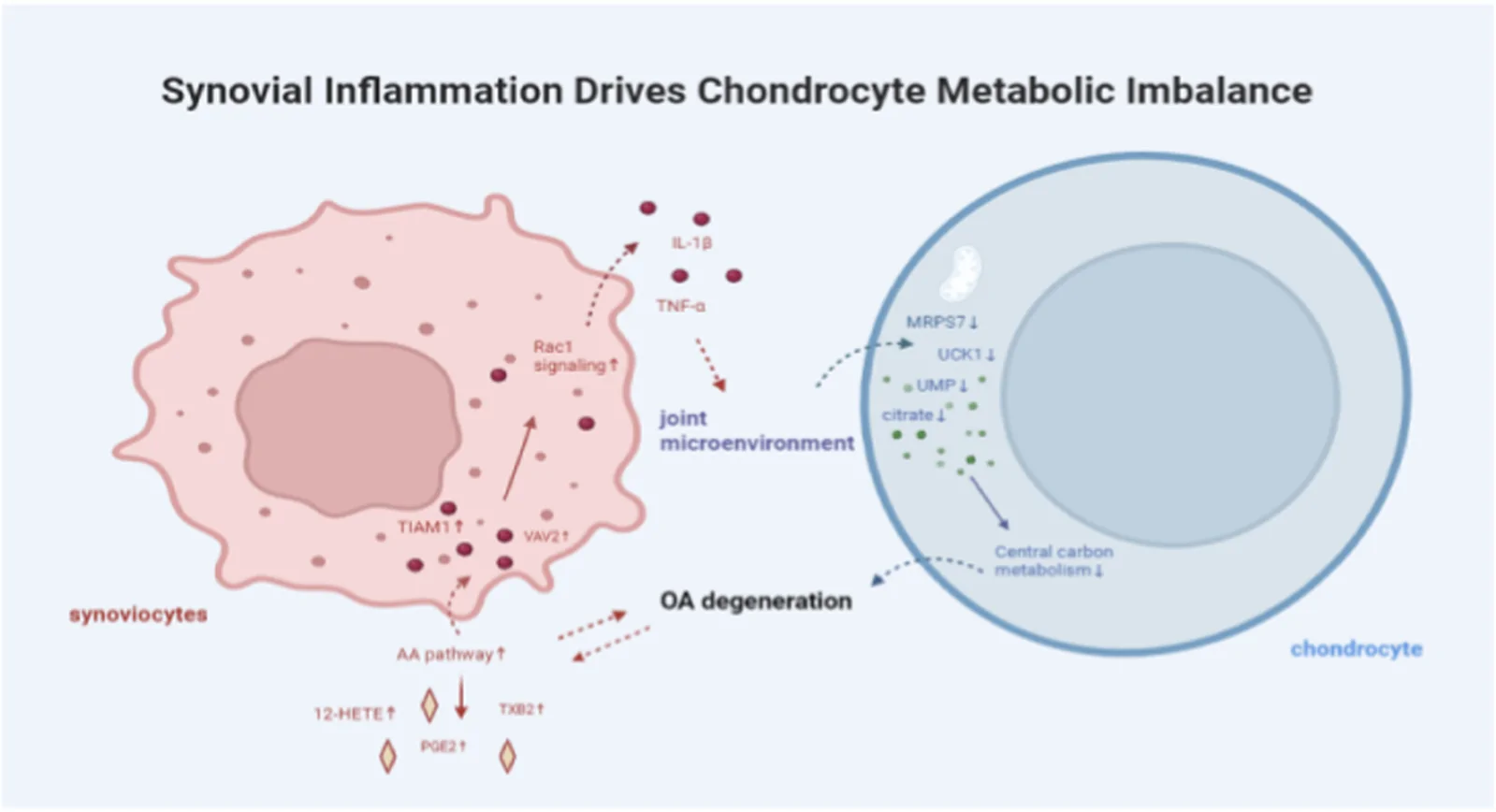
Hypothetical model of synovial inflammatory activation and chondrocyte metabolic imbalance. Based on the proteomic, metabolomic, and protein–metabolite integrated analyses, we propose a potential link between synovial inflammatory activation and chondrocyte metabolic imbalance in OA. In OA synovium, enhanced TIAM1/VAV2/Rac1-related signaling, activation of arachidonic acid metabolism, and increased inflammatory lipid mediators such as PGE2, TXB2, and 12-HETE suggest an inflammatory–metabolic activation state. These changes may alter the joint microenvironment and may be associated with impaired mitochondrial/translational function and abnormal central carbon metabolism in chondrocytes. In OA cartilage, decreased MRPS7, UCK1, and citrate suggest insufficient energy supply and impaired metabolic homeostasis. This figure summarizes a potential association rather than a proven causal relationship.

This study has several limitations. Utilizing a cross-sectional design, it performed a multi-omics analysis of paired synovial and cartilage tissues from OA patients and control individuals. Although proteomics and metabolomics demonstrated a high degree of consistency in their functional direction, the temporal sequence and causal relationships of molecular events in the synovial and cartilage tissues could not be directly analyzed. In addition, the cross-tissue integrative model of synovium and cartilage proposed in this study is mainly based on differentially expressed proteins, differential metabolites, pathway enrichment, protein–metabolite integrated analysis, and functional annotation. It represents a functional integration and hypothetical summary of the multi-omics results, rather than a strict paired cross-tissue correlation analysis or causal inference. Therefore, although our results suggest functional consistency and complementarity between synovial inflammatory–lipid metabolic activation and restricted cartilage energy metabolism, the conclusion that synovial molecular changes directly drive cartilage metabolic abnormalities still requires further validation. On the other hand, Rac1 was examined in this study as a downstream pathway-related effector of TIAM1. The Western blot results reflect changes in total Rac1 protein abundance and do not directly indicate enhanced GTP-Rac1 activity. Future studies should incorporate Rac1 activity assays, synovial cell functional experiments, chondrocyte co-culture models, and analyses of extracellular vesicle-mediated communication to further validate the functional links among TIAM1/Rac1-related signaling, synovial inflammatory lipid mediators, and restricted cartilage metabolism. Additionally, the relatively small sample size presents another limitation. This constraint primarily arises from the challenges associated with sample acquisition, which typically depend on large-scale surgical procedures such as tibial plateau fractures and lower limb amputations, rather than routine clinical practices. Consequently, related studies often face limitations in sample size. Consequently, this study could not perform an association analysis between omics characteristics and clinical severity indicators, such as the Kellgren-Lawrence classification, WOMAC score, or pain VAS score. Such analyses are instrumental in assessing the potential value of biomarkers for disease stratification and translational applications. On the other hand, although these control samples showed no obvious cartilage degeneration or synovial inflammation on preoperative imaging and intraoperative assessment, and inflammatory, metabolic, and immune system diseases had been excluded, the primary injury, surgical indications, and local tissue status may still have affected protein expression and metabolic profiles. Therefore, the heterogeneity of the control sources may have had a potential impact on some molecular differences, and the related findings still need to be further validated in more strictly matched independent cohorts. In addition, there were certain differences in age and body mass index (BMI) between the OA and control groups. Age and BMI have been shown to affect systemic inflammatory responses and metabolic status, and their potential confounding effects should therefore be considered when interpreting the results. However, due to the limited sample size, this study was unable to perform sufficient multivariable adjustment models or sensitivity analyses to systematically exclude the potential effects of age, BMI, and other factors on inflammatory status and metabolic profiles. Therefore, results involving inflammatory responses, lipid metabolism, and energy metabolism should be interpreted with caution in the above clinical context. Future studies with larger sample sizes, multicenter cohorts, and more strictly matched populations are needed for further validation. Nevertheless, this study meticulously selected representative OA samples and employed rigorous statistical methods to minimize the influence of random factors, thereby improving the reliability of the findings.

Although this study employs proteomics and metabolomics analysis at the tissue level to systematically elucidate the overall molecular characteristics of the synovial membrane and cartilage in the OA state, it remains limited in its ability to analyze the relative contributions of specific cell types to the observed molecular changes. Given the substantial heterogeneity in cellular composition of both synovial and cartilage tissues, we are currently employing high-resolution techniques, including single-cell transcriptomics, to elucidate the cellular sources and interactions of key signaling pathways and metabolic reprogramming at the level of individual cell types. Consequently, the cross-omics integration results presented in this study at the organizational level provide critical insights and a theoretical foundation for future single-cell investigations.

## Conclusion

This study presents an integrated analysis of proteomics and metabolomics conducted on paired synovial and cartilage tissues. The findings indicate that different joint tissues in OA exhibit opposing yet functionally interconnected changes at the molecular level. Specifically, signals related to synovial inflammation are activated, accompanied by an increase in metabolic output, while energy and anabolic metabolism in cartilage are generally restricted. This research offers a novel perspective on metabolic-signal integration, enhancing our understanding of the cross-tissue interactions between synovium and cartilage in OA at a systemic level. The identified metabolic abnormalities and their upstream regulatory factors may hold potential for interventions aimed at delaying the progression of OA.

## Data Availability

The untargeted metabolomics data generated in this study have been deposited in the MetaboLights repository under accession number MTBLS14917 (https://www.ebi.ac.uk/metabolights/MTBLS14917/). The DIA proteomics dataset has been deposited in the PRIDE database under accession number PXD080555 (https://www.ebi.ac.uk/pride/archive/projects/PXD080555). Other data supporting the findings of this study are available within the article and its Supplementary Materials.
