# Supplementary material for: Multi-omics analysis unveils inflammatory and metabolic alterations between the synovium and cartilage in patients with knee osteoarthritis
Source: Front Mol Biosci. 2026 Sep 22;13:1855139. doi: 10.3389/fmolb.2026.1855139 (PMC13639140; doi:10.3389/fmolb.2026.1855139)
Supplement: Supplementary file 1 [file Supplementaryfile1.docx]

Supplementary Figure 1A.Principal component analysis (PCA) of synovial tissue proteomics data showing the distribution of OA (IS) and control (NS) samples. PC1 explains 23.48% of the variance, demonstrating clear separation between the two groups.

Supplementary Figure 1B.Heatmap of the top 100 differentially expressed proteins in synovial tissue, illustrating the expression patterns between OA (IS) and control (NS) groups.

Supplementary Figure 2A.Principal component analysis (PCA) of cartilage tissue proteomics data showing the distribution of OA (DC) and control (NC) samples, indicating significant differences between the two groups.

Supplementary Figure 2B.Heatmap of the top 100 differentially expressed proteins in cartilage tissue, showing distinct expression profiles between OA (DC) and control (NC) groups.

Supplementary Figure 3A.Pearson correlation heatmap of quality control (QC) samples, demonstrating high reproducibility and stability of the LC-MS/MS platform (all correlation coefficients ≥ 0.988).

Supplementary Figure 3B.PLS-DA score plot showing the separation between OA and control groups based on metabolomics data.

Supplementary Figure 3C.PLS-DA validation plot with 200 permutation tests, confirming the reliability of the model (Q² intercept < 0, R² > Q²).

Supplementary Figure 3D.ROC curve analysis of differential metabolites in synovial tissue, showing the discriminatory power of individual metabolites (AUC: 0.91-0.94) and the combined logistic regression model (AUC: 0.958).

Supplementary Figure 3E.ROC curve analysis of differential metabolites in cartilage tissue, demonstrating their potential as biomarkers for distinguishing OA from control samples.

Supplementary Figure 3F.Heatmap of the top 30 differentially abundant metabolites in synovial tissue, showing relative abundance between OA and control groups.

Supplementary Figure 3G. Heatmap of the top 30 differentially abundant metabolites in cartilage tissue, illustrating distinct metabolic profiles between OA and control groups.
